# The multicultural evolution of beauty in facial surgery^☆^

**DOI:** 10.1016/j.bjorl.2017.04.005

**Published:** 2017-05-02

**Authors:** Eric W. Cerrati, J. Regan Thomas

**Affiliations:** a University of Illinois at Chicago, Department of Otolaryngology-Head and Neck Surgery, Division of Facial Plastic & Reconstructive Surgery, Chicago, USA; b University of Illinois at Chicago, Department of Otolaryngology-Head and Neck Surgery, Chicago, USA

The concept of facial beauty has been defined in a variety of ways dating back to ancient times, and while the definition continues to develop, it has become clear that beauty crosses ethnic boundaries and has a significant cultural and economic impact. Subconsciously, beauty is perceived by humans as a sign of favorable genes and increased fertility, both of which play a role in mate selection. As a result, perceived attractive features that are subconsciously selected evolve much more quickly than other naturally selected characteristics. Additionally, the beautiful are more likely to get better grades in school, to be hired for a job, to receive higher salaries, and to be viewed as nicer, smarter and healthier.^1^ While beauty was once stated to be “in the eye of the beholder”, more recent studies have suggested that beauty is an objective, quantifiable quality.

The ancient Greeks began the quest for a universal standard of beauty and believed it was represented by the “golden ratio” also known as “*phi*,” which was thought to represent perfect harmony.^1^, ^2^, ^3^ In nature, the ratio appears in the spiral of seashells, in the growth rate of the human mandible, and in the DNA antihelix. Examples of its application include Egyptian art and architecture, the Fibonacci sequence, and geometric shapes such as the pentagon and decagon. Many still believe that phi corresponds to facial beauty as well.^3^ However, others have found it to be inexact. For example, Marquardt created an “ideal” facial standard based off of phi, and not only did it apply poorly to people of non-European/Caucasian descent but it also masculinized Caucasian women.^4^

The concept of beauty as a formula continued to evolve with the artists of the Renaissance period. Through Da Vinci and his contemporaries, the neoclassical ideals were largely based on phi. The art anatomists of the 17th and 19th centuries propagated these new standards into the medical field, which created a “universal” definition of beauty for the period.^2^ While these ideals continue today to have a strong influence on facial analysis and serve as a guideline for surgical planning, research has shown that these ideals still do not apply cross-culturally.

Despite the inability to universally quantify beauty, researchers have found that there is a consensus on rating attractiveness across sexual orientations, ethnic groups, and ages. Studies have shown that diverse populations agree on who is and is not attractive. Additionally, even infants have an innate preference toward attractive faces.^1^ Certain conceptions of facial beauty or attractiveness may be everlasting. In 2006, Bashour researched and challenged each of the four concepts. He concluded that subjective attractiveness comprises only a small percentage of personal preference over a much larger biological objective assessment of attractiveness.^5^

The four concepts of facial beauty include symmetry, averageness, youthfulness, and sexual dimorphism. The first concept of symmetry is believed to represent a high quality of development. A symmetric face reflects a person's phenotypic and genetic condition giving him or her an advantage in sexual competition. Averageness, the second concept, is informed by the Darwinian theory that evolutionary pressures function against the extremes of the population. As a result, humans innately appreciate that averageness represents genetic heterozygosity and a greater resistance to disease. The third concept is youthfulness. Neonatal features, such as large eyes and a small nose, are believed to suggest desirable qualities of youthful liveliness, open-mindedness and affability. As a person ages and demonstrates soft tissue descent, the face deviates from the phi standard, resulting in a decrease in attractiveness. In addition, the human brain interprets the physical changes of aging as a decrease in fertility. The fourth and last concept of beauty is sexual dimorphism, which is defined as a phenotypic difference between males and females. For females, increased estrogen leads to the development of secondary characteristics that suggest a fertile host and a reproductive advantage. These include a thin jaw, small chin, large widely spaced eyes, small nose, high cheekbones, and plump lips. On the contrary, desirable physical features in men are those that signify high testosterone levels, such as prominent chins, square jaws, deep-set eyes, thin lips, heavy brows and abundant hair.^1^, ^2^

Although attractiveness can be agreed upon cross-culturally, each ethnicity has unique features that are factored into its definition of “averageness.” For the facial plastic surgeon, these unique features must be respected and embraced in order to create a harmonious and elegant result that meets the criteria of beauty and attractiveness. As a result, the neoclassical ideals may not serve as accurate guidelines in non-Caucasian patients. Specifically in rhinoplasty, distinct anatomic differences exist between the leptorrhine nose seen in Caucasians, the platyrrhine nose seen in African and Asian populations, and the mesorrhine nose seen in Latin American populations.^1^ Patients frequently want to preserve their cultural identity, so it is paramount that the surgeon clearly distinguishes these goals preoperatively.

Today's typical facial plastic surgery practice is becoming increasingly multicultural. The globalized modern society has played a significant role in the perception of beauty. Economic mobility coupled with an increase in interracial couples has blurred the lines of ethnic identity, and the resulting esthetically unique and beautiful outcomes do not allow patients to be characterized as fitting a narrow mold with predictable desires.^1^ The classic principles of beauty including phi, symmetry, averageness, youthfulness, and sexual dimorphism can still be applied as guidelines, but the surgeon must incorporate a broader outlook on facial analysis and surgical techniques. The importance of identifying patients’ ethnic identities cannot be underemphasized as patients may want to erase, preserve, modify, or even enhance those specific inherent traits.

Furthermore, cosmetic surgery continues to become increasingly desirable and socially acceptable. The amplified attention and interest can be credited to its exposure in reality television, social media, and surgical documentaries. The increased demand and the rising population diversity ensure that each patient will present with a unique background and cosmetic objective. The surgeon should assist patients to arrive at a goal that is harmonious with their face giving a timeless, attractive result rather than be swayed by the development of a fashion trend. The proper guidance, insight, and ethical control distinguish the surgeon from a technician. More importantly, these qualities preserve the integrity of the field of facial plastic surgery.

While facial modifications can have a tremendous impact on patients’ lives, the planned result should not venture too far from the concepts of facial beauty that have defined the field since its creation. Digital photography along with computer imaging has aided with preoperative assessments in an effort to confirm the surgeon and the patient have the same esthetic goals. Technology will continue to improve to facilitate this initial conversation. As society evolves so must our understanding of beauty along with our attempt to surgically define it.

## Conflicts of interest

The authors declare no conflicts of interest.
